# Favourable prognosis of trigeminal neuralgia when enrolled in a multidisciplinary management program - a two-year prospective real-life study

**DOI:** 10.1186/s10194-019-0973-4

**Published:** 2019-03-04

**Authors:** Tone Bruvik Heinskou, Stine Maarbjerg, Frauke Wolfram, Per Rochat, Jannick Brennum, Jes Olesen, Lars Bendtsen

**Affiliations:** 1 Danish Headache Center, Department of Neurology, Rigshospitalet-Glostrup, Faculty of Health and Medical Sciences, University of Copenhagen, Valdemar Hansens Vej 5, Rigshospitalet-Glostrup, DK-2600 Glostrup, Denmark; 2 Department of Diagnostics, Herlev Hospital, Herlev, Denmark; 3 Department of Neurosurgery, Rigshospitalet-Blegdamsvej, University of Copenhagen, Copenhagen, Denmark

**Keywords:** Individualized drug treatment, Outcome, Natural history, Real-life study, Multidisciplinary management, Observational study

## Abstract

**Background:**

Prognosis of medically treated trigeminal neuralgia patients is assumed to be poor, but the evidence is lacking. Thus, prospective real-life studies of medical management of trigeminal neuralgia are warranted.

**Methods:**

This was an observational study. Patients were consecutively enrolled in a structured management program at a specialist centre for facial pain. Optimisation of medical treatment, physiotherapy, psychotherapy, and advice from trained nurses, were parts of the program. Medically intractable patients were referred for neurosurgery. Data-collection was prospective using standardised schemes and patient surveys. The aim was to describe the two-year outcome of medical treatment at the specialist centre. The primary outcome was a 50% reduction in the overall burden of pain according to a Numerical Rating Scale (NRS) after two years.

**Results:**

A total of 186 primary TN patients were enrolled in the program of which 103 patients remained medically managed and completed the two-year follow-up. Fifty patients were treated surgically within the first two years of follow-up. Half of the medically managed patients (53 (51%)), had more than a 50% reduction in the overall burden of pain over the two-year period. The overall burden of pain on NRS decreased from mean 5.34 to 3.00, *p* < 0.01. There was no significant association between primary outcome and sex, depression and/or anxiety, concomitant persistent pain, or neurovascular contact with morphological changes of the trigeminal nerve.

**Conclusions:**

Patients with trigeminal neuralgia improve over a two-year period when enrolled in a structured medical management program. Optimisation of drug treatment, continuous advice and education and support by the multidisciplinary team, referral of the medically intractable patients for surgery or the natural history of the disease, can be some of the reasons for the improvement. The favourable prognosis provides hope and optimism for medically managed TN patients.

**Trial registration:**

Current study was observational, and patients were offered standard clinical care and laboratory workups according to current American Academy of Neurology and European Federation of Neurological Societies treatment guidelines. The study has been registered at ClincalTrials.gov. ID: NCT03838393.

**Electronic supplementary material:**

The online version of this article (10.1186/s10194-019-0973-4) contains supplementary material, which is available to authorized users.

## Introduction

Trigeminal neuralgia (TN) is a facial pain disorder characterised by recurrent paroxysms of severe unilateral pain distributed in one or more branches of the trigeminal nerve [1]. The natural history of TN is commonly assumed to be progressive and the prognosis of the disease to be poor with slow deterioration over time [1–3]. However, the findings in more recent studies do not confirm the progressive nature of the disease [4–7] and the natural history is not fully elucidated. Several studies describe a good prognosis for patients treated neurosurgically [8–10]. However, only a few high-quality cohort studies have investigated the prognosis of TN when treated medically [4, 7].

Considerable expertise is necessary for proper medical management of TN because the drugs are unspecific, mostly antiepileptics, and often have to be administered in a high dose to control the pain [11]. Furthermore, the scientific evidence for drug treatment of TN is weak [11, 12], and the treatment is often hampered by insufficient effect, pharmacological interaction, and side-effects [12, 13].

Randomised controlled trials are the gold standard for assessment of efficacy and tolerability of drug treatment [14]. Such trials are usually short-term trials enrolling highly selected patients and thus may both over- and underestimate the efficacy of drug treatment in a real-life setting [15, 16]. Moreover, these trials do not take other important factors into consideration when it comes to the optimal management of TN over time. This includes finding the suitable drug and dose for the individual patient, continuous optimisation of drug treatment according to disease activity and side-effects [11], and the value of education and continuous advice and support. Thus, to investigate the value of the various treatment options and to describe the prognosis of the disease when medically managed, real-life studies are warranted [17].

This study aimed to provide evidence concerning the real-life efficacy of medical management of TN when directed by specialists. Additionally, the aim was to inspire other neurological centres to improve their TN management programs to ensure the best possible care for this patient group. We hypothesised that the two-year prognosis in a group of medically managed TN patients enrolled in a structured multidisciplinary management program was favourable, defined as a 50% reduction of the overall burden of pain over a two-year period.

## Material and methods

This study was a prospective, observational study of TN patients enrolled in a structured multidisciplinary management program at the Danish Headache Center (DHC), as previously described in detail [18]. The program was introduced in May 2012 and was based on the standard clinical care and laboratory workups according to current guidelines [11]. The DHC is a national centre of excellence that treats approximately 100 new TN patients per year.

### Description of the cohort

Newly referred TN patients seen between May 2012 and December 2015 were enrolled consecutively and followed-up systematically. Inclusion criteria were: recordings of the intensity of pain at enrolment of the programme (baseline recording), a two-year assessment (end-point recording), and effect and side-effects of drug treatment (at baseline and endpoint). The exclusion criteria were: initiation of medical treatment at the DHC before May 2012, neurosurgical treatment of TN within the two-year follow-up, and incomplete two-year follow-up. The number of patients enrolled in the inclusion period determined the sample size.

The diagnostic criteria used in this study were the beta version of the 3rd edition of the International Classification of Headache Disorders (ICHD-3-beta) [19]. Until the publication of IHCD-3-beta, ICHD 2 [20] criteria were used. According to the ICHD-3 classification [1], this study included patients with both idiopathic and classical TN; in this paper termed *primary* TN.

### Outpatient visits

At enrolment, all medically managed patients were offered five fixed visits within a two-year period. Follow-up regime has previously been described in detail [18]. In short, the patients visits were: Initial visit (clinical interview, clinical and paraclinical examinations, diagnosis and treatment, information concerning pharmacological, non-pharmacological and neurosurgical treatment options (verbal and written)); and follow-up visits at approximately 3 (2–4) months, 9 (7–11) months, 15 (12–18) months and 24 (21–28) months after their initial visit. At the follow-up visits, the treating physician evaluated treatment effect and, in line with the international guidelines [11], encouraged patients to adjust the dosage of medication according to their burden of pain. The need for referral to neurosurgical treatment was evaluated continuously. The neurosurgical options were microvascular decompression, balloon compression and glycerol blockade. If patients were neurosurgically treated they followed a slightly different follow-up regime, as described previously [18]. Between the follow-up visits, the patients could call a trained staff-nurse, with questions concerning dose adjustment, change of drugs, side-effects or other concerns regarding TN. Therapy sessions with a trained DHC psychologist and/or with a physiotherapist where offered if needed. Consultations with a neurosurgeon were also offered if the patients wanted in-depth information about possibilities for surgery.

At the initial visit, a routine physiological and neurological examination was performed with a special focus on the trigeminal sensory function, as previously described in detail [18].

### Neuroimaging

A 3.0 Tesla MRI scan was conducted according to a TN protocol [21]. The TN protocol was pre-defined for the trigeminal nerve to exclude secondary cause of TN and to ensure evaluation of the neurovascular relations, degree, localisation, and type of neurovascular contact if any. Patients that suffered from claustrophobia, severe obesity or had a pacemaker were offered an MRI scan in an open 1.5 Tesla scanner. These scans were without the predefined TN protocol. All scans were evaluated by the same experienced neuroradiologist (FW), who was blinded to the pain side.

### Data collection

Assessments were conducted by a neurologist or highly trained fellow in neurology at the DHC at all visits. At the initial visit, a standardised purpose-built semi-structured interview was performed by a highly-trained fellow in neurology, supervised by a senior neurologist. At the subsequent follow-up visits, the patients were assessed by the same senior neurologist. For a detailed description of the semi-structured interview see Additional file 1. At the initial interview and the assessments, the following was evaluated; a) the overall burden of pain was recorded on a Numeric Rating Scale (NRS). Overall burden of pain was defined as the patient’s self-perceived burden of pain (global impression of pain intensity and frequency of attacks) on average on a scale from 0 to 10, over the last month; b) effect of current drug treatment was according to the Barrow Neurological Institute pain scale (BNI) [22], assessed over the last month; c) daily dosage and side-effects of current TN drugs. The evaluations from the assessments were given verbally by the patients and registered in standardized schemes filled out by the physician.

A self-complete patient survey was mailed to the patients two years after their initial visit. If the patients did not return the questionnaire, a reminder was mailed to them again 26–27 months after enrollment. If they did not return the second questionnaire, data was registered as missing. The survey included 21 qualitative and quantitative questions, corresponding the assessment schemes, concerning; overall burden of pain, pain quality, current treatment and satisfaction with treatment. Satisfaction levels were registered on a 7-point Likert Scale, anchored at 1 = very dissatisfied and 7 = very satisfied. For the analysis, the patients were considered as satisfied if 5–7 was reported on the scale, while the patients were regarded as dissatisfied if 1–3 was reported on the scale. The patients were regarded as neither satisfied or dissatisfied if they reported 4 on the scale. See Additional file 2 for a detailed description.

Data from the control schemes and questionnaires were used to evaluate the outcome. If there was disagreement between the two-year follow-up assessment and the patient survey, data from the follow-up scheme was reported in this study.

### Outcome measures

The primary outcome was a good outcome according to the overall burden of pain NRS, i.e. the number of patients who had a reduction of minimum 50% on the NRS two years after enrolment in the management program compared with baseline. NRS was anchored at 0 = *no burden of pain* and 10 = *worst possible burden of pain*. To grade the burden of pain on the NRS, the scale was subcategorised into NRS 0 = no burden of pain, NRS 1–4 = mild burden of pain, NRS 5–7 = moderate burden of pain, NRS 8–10 = severe burden of pain [23]. If the patient did not take any medication and reported NRS = 0, the pain was regarded as in remission.

The secondary outcomes were; a) poor outcome defined as no reduction of the overall burden of pain NRS over the two-year follow-up period. NRS was anchored as described above, and b) good treatment effect according to BNI defined as the number of patients who reported BNI level 0, I or II two years after enrolment. BNI I = Very good effect: *No pain*, BNI II = Good effect: *Occasional pain that does not, or only occasionally, reduce my quality of life,* BNI III = Limited effect: *Daily pain with a moderate reduction of my quality of life,* BNI IV = Insufficient effect: *Daily episodes with severe pain which significantly reduce my quality of life.* If patients were not taking any TN drugs, BNI was registered as BNI = 0. Patients who were not taking any medication (BNI = 0) were subdivided into two groups a) patients in remission b) patients without medical treatment but not pain-free.

### Statistical analyses

Continuous and ranked data are summarized by descriptive statistics. Categorical variables are presented with frequency distributions (N, %) and with 95% confidence limits (Cl). McNemar’s, Chi-square and Fisher’s test were used to assess associations of categorical variables. Nonparametric testing (Wilcoxon signed-rank test) was used to determine the significance of differences in reported outcome measures, e.g. NRS and BNI, between baseline and endpoint assessments.

A multivariate logistic regression analysis with backward stepwise selection was used to test for associations between a subset of predefined variables and good outcome. The same multivariate logistic regression analysis was also conducted to test for associations between a subset of predefined variables and poor outcome. Variables were retained in the regression model if a significant association (*p* < 0.05) was found. Variables with no significant association with outcome were excluded sequentially. The predefined independent variables were sex (male vs. female), anxiety and/or depression (yes vs. no), disease duration (≥ 5 years vs. < 5 years), degree of neurovascular contact at MRI (with vs. without morphological changes of the trigeminal nerve) and pain quality (with concomitant persistent pain vs. purely paroxysmal pain). In a previous study, which included a subset of the patients also included in the current study, we found a strong association between the male sex and a neurovascular contact with morphological changes [21]. Thus, the multivariate logistic regression model was controlled for this confounding interaction.

To test for associations between a good outcome and the variables; suffering from other headaches, suffering from other chronic pain conditions, hypertension, response to sodium-channel blockers, and findings of sensory abnormalities of the trigeminal nerve found at the neurological examination, we used 2 × 2 contingency chi-square test or Fisher’s exact test.

If more than 10% of the clinical data from the semi-structured interview or two-year assessments were missing, the patient was excluded from the analyses. Missing data were considered missing at random, based on the assumption that missingness was independent of the demography, treatment, and pain level. *P*-values are reported as two-tailed with a level of significance of 5%. Analyses were carried out using SAS 9.4 (SAS Institute Inc., Cary, NC, USA).

## Results

**Fig. 1:**
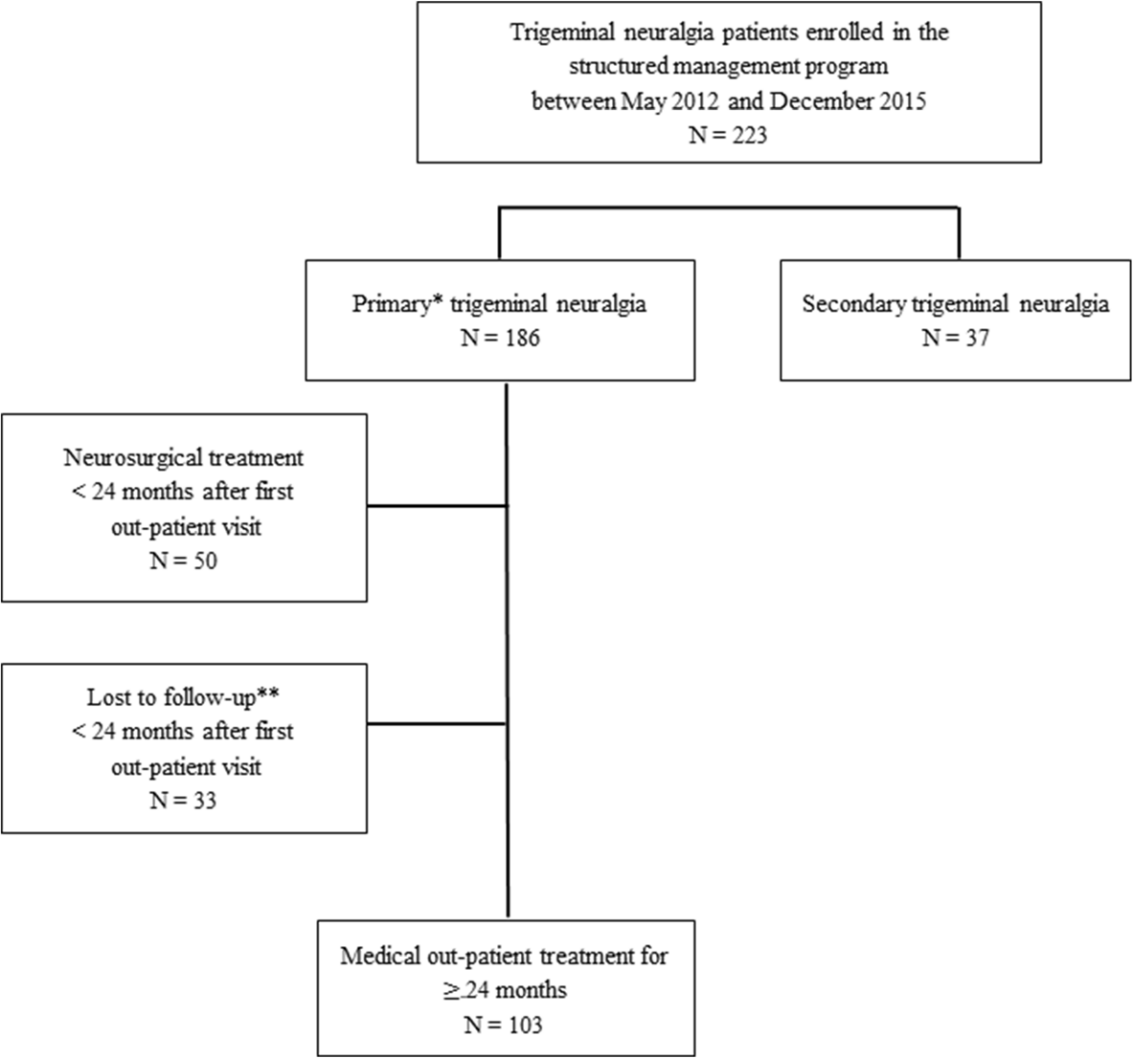
Flowchart of included patients

From May 2012 to December 2015, 223 patients were enrolled in the management program. A total of 186 patients were diagnosed with primary TN and 37 patients with secondary TN (Fig. 1). Of the 186 primary TN patients, 50 patients (27%) were treated surgically, i.e. underwent either MVD, glycerol blockade or balloon compression, within the first two years of their follow-up. The mean time from the initial visit to surgery was 217 days, range 16–710 days. The reasons for discontinuation of medical treatment was due to lack of efficacy or intolerable side effects. Thirty-three patients were lost to follow-up or excluded from the study. A total of 103 patients, (72 women and 31 men) were included in the study.

The male: female ratio was 1:1.75. The mean age at onset was 52.5, range 12–81 years. The mean duration of the disease at the initial visit was 4.4 years, range 0–32 years. The full demographical data, including the data of the patients that were referred for neurosurgery < 24 months after their initial visit, is shown in Table 1.

**Table 1:** Demographics and comorbidities

Characteristic	24 months medical follow-up	Surgical treatment < 24 months after 1st outpatient visit	*p*-value
	N (%) 95% Cl mean	N (%) 95% Cl mean	
Demographics			
Total number of patients	103 (67)	50 (33)	
Women	72 (70) 60–78	28 (56) 21–34	0.13^a^
Current age	57.2 54.3–60.1	61.8 57.8–65.8	0.069^b^
Age at onset, mean, years	52.5 49.7–55.3	54.0 49.8–58.2	0.58^b^
Disease duration, mean, years	4.4 3.4–5.5	6.2 4.7–7.8	0.10^b^
Clinical characteristics			
Right-sided pain	60 (58) 49–68	26 (52) 37–66	0.42^a^
Bilateral pain	8 (7) 3–15	0 (0) n/a	0.055^c^
Concomitant persistent pain	55 (53) 43–63	26 (52) 37–66	0.87^a^
Localization of pain			
V1	3 (3) 0–9	1 (2) 0–10	1.00^c^
V2	19 (18) 2–26	6 (12) 5–24	0.44^a^
V3	20 (19) 13–28	15 (30) 19–44	0.21^a^
V1+ 2	9 (9) 5–16	5 (10) 4–22	0.80^a^
V2 + 3	37 (36) 27–46	15 (30) 17–45	0.59^a^
V1+ 2+ 3	15 (15) 9–23	8 (16) 8–29	0.82^a^
Comorbidities			
Hypertension	32 (31) 22–40	15 (30) 17–45	0.89^a^
Other chronic pain conditions	11 (11) 6–17	9 (18) 9–31	0.17^a^
Depression and/or anxiety	15 (15) 8–23	7 (14) 6–29	0.89^a^
Cardiovascular disease	8 (7) 4–14	8 (16) 7–29	0.20^a^
Tension type headache, migraine or cluster headache	24 (22) 15–31	7 (14) 6–29	0.26^a^

Demographics of patients with at least 24 months medical follow-up and patients who were referred for surgery < 24 months after initial outpatient visit. ^a^Chi square ^b^ Unpaired t-test ^c^ Fishers exact test. n/a = not applicable. *P* < 0.05 was considered significant

### Primary outcome measure

The overall burden of pain was reduced from mean NRS 5.34 (Cl 4.79–5.90) to 3.00 (Cl 2.47–3.53), *p* < 0.001. Two years after enrolment 53 patients (51%) had a good outcome, i.e. a minimum reduction of 50% in the overall burden of pain on NRS, compared with baseline. Significantly more patients reported no overall burden of pain (NRS 0) (16 vs. two patients) and mild burden of pain (NRS 1–4) (63 vs. 38 patients) at their two-year assessment compared to baseline (*p* < 0.001 and *p* = 0.002, respectively). A severe burden of pain (NRS 8–10) was reported by fewer patients (7 vs. 25 patients) at their two-year assessment compared to baseline (*p* < 0.001). The number of patients with a moderate burden of pain was unchanged (38 patients vs. 22 patients (*p* = 0.06)) (Table 2).

**Table 2:** Changes in overall burden of pain over the last month according to Numeric Rating Scale

Overall burden of pain (NRS)	Grading of overall burden of pain	Overall burden of pain initial assessment *N* (%)	Mean overall burden of pain - initial assessment (Cl)	Mean change in overall burden of pain at two-year assessment (Cl)	*p*-value^#^
0	No burden of pain	2 (2)	0	0.5 (−5.85–6.85)	0.5
1	Mild burden of pain	10 (10)	2.58 (2.18–2.98)	−2.32 (−3.27- -1.36)	0.003
2		9 (9)			
3		6 (6)			
4		13 (13)			
5	Moderate burden of pain	16 (16)	5.92 (5.63–6.21)	−0.97 (−1.63 - - 0.32)	< 0.001
6		9 (9)			
7		13 (13)			
8	Severe burden of pain	10 (10)	9.2 (8.72–9.52)	−5.60 (−6.83 - - 4.37)	< 0.001
9		2 (2)			
10		13 (13)			

*N* = 103. *NRS* = Numeric Rating Scale, ^#^Wilcoxon Signed Rank test. Cl = 95% confidence limits. *P* < 0.05 was considered significant

### Secondary outcome measures

A poor outcome was reported by 33 (32%), of whom 24 patients (23%) reported no changes in the overall burden of pain, and nine patients (9%) experienced a worsening of the overall burden of pain, over time.

Of the 16 patients who reported moderate or severe burden of pain at their two-year assessment, four patients underwent microvascular decompression after the two-year assessment. Seven patients were satisfied with their drug treatment, despite the increase of burden of pain during the follow-up. Two patients were referred to neurosurgical consultation but wanted time to consider the pros and cons for surgery. One patient had co-existing severe rhinitis at the time of assessment that might have worsened the TN. Thus, she was not considered a candidate for surgery, at the time. The reason for not undergoing neurosurgery was unknown in two patients.

At the two-year follow-up, there was a median improvement of 1 on the BNI scale, (*p* < 0.001), reduced from a median of 2 (SD 1.31) to 1 (SD 1.16) on the BNI scale. Significantly more patients reported a good treatment effect according to BNI (BNI 0, I or II) at the two-year assessment (85 patients (83%)), compared with baseline (53 patients (53%)), (*p* < 0.001) (Table 3).

**Table 3:** Barrow Neurological Institute pain intensity score at baseline and at two-year assessment

Score	Pain description	Initial assessment *N* (%)	Two-year assessment *N* (%)	Median change of BNI	*p*-value^#^
BNI 0	No medication needed at the time of assessment	16 (16) *	29 (28) **	0	0.25
BNI I	Very good effect: *No pain*	17 (17)	28 (27)	0	0.30
BNI II	Good effect: o*ccasional pain that does not, or only occasionally, reduce my quality of life*	20 (19)	28 (27)	- 1	0.078
BNI III	Limited effect: *Daily pain with a moderate reduction of my quality of life*	35 (34)	13 (13)	- 1	< 0.001
BNI IV	Insufficient effect: *Daily episodes with severe pain which significantly reduce my quality of life*	15 (15)	5 (5)	−2	< 0.001

N = 103. *BNI* = Barrow Neurological Institute pain intensity score. ^#^ Wilcoxon Signed Rank test. *Two patients were in remission (no pain) while 18 were reporting pain on the VNRS (not pain-free). ** Nineteen patients were in remission (no pain) while 10 were reporting pain on the VNRS (not pain-free). Thirty-seven patients (36%) reported unchanged BNI level, while 14 (14%) patients had a worsening in BNI over the two-year follow-up period. *P* < 0.05 was considered significant

### Association between clinical characteristics and outcome

Patients with at least five years disease duration had 2.5 times higher odds of a good two-year outcome compared to patients with a disease duration of 0–4 years (Cl 1.06–5.79), *p* = 0.036. The regression model did not show any significant association between good outcome and sex, morphological changes of the trigeminal nerve on the MRI, depression and/or anxiety, or concomitant persistent pain (Table 4). Post hoc analysis showed that suffering from other chronic pain diseases was associated with a reduced chance of good outcome, (OR 0.13, Cl 0.03–0.63), *p* = 0.009. Post hoc analysis showed no association between good outcome and the following clinical characteristics: bilateral pain (*p* = 0.48); suffering from other headaches (*p* = 0.90); hypertension, (*p* = 0.66); depression/anxiety (*p* = 0.11) or age < 40 at onset (*p* = 0.88). (Additional file 3). Poor outcome was not associated with any of the before mentioned clinical variables.

**Table 4:** Associations between clinical characteristics and good outcome

Prognostic variable	OR	95% Cl	*p*-value
Disease duration (≥ 5 years vs. < 5 years)	2.5	1.1–6.0	0.036
Anxiety and/or depression (yes vs. no)	0.4	0.1–1.2	0.084
Neurovascular contact with morphological changes found on MRI (yes vs. no)	0.8	0.3–2.0	0.68
Sex (man vs. woman)	1.2	0.5–3.3	0.68
Purely paroxysmal pain (yes vs. no)	0.9	0.4–2.3	0.88

N = 103 TN patients, of whom 53 had a good outcome. Good outcome = 50% reduction of the overall burden of pain according to NRS. The analysis of the association between the prognostic variable and good outcome was done by multiple logistic regression with back-wards elimination. *p*-values are reported as two-tailed with a level of significance of 5%. OR = odds ratio; Cl = 95% confidence limits

### Clinical characteristics and drug treatment

The clinical characteristics of the medically treated patients did not differ from the characteristics of the patients who were referred to neurosurgery before their two-year follow-up (Table 1). The neuroimaging findings of the trigeminal nerve were also similar in the two groups (Table 5).

**Table 5:** Baseline neuroimaging findings

MRI findings				
		24 months medical follow-up	Surgical treatment < 24 months after 1st outpatient visit	*P*-value
		*N* = 95*	*N* = 50	
		N (%) 95% Cl	N (%) 95% Cl	
Grade	Neurovascular contact *with* morphological changes** (Classical trigeminal neuralgia)	37 (39) 28–48	25 (50) 36–65	0.27
	Neurovascular contact *without* morphological changes (Idiopathic trigeminal neuralgia)	44 (46) 36–57	22 (44) 30–59	0.93
	*No* neurovascular contact (Idiopathic trigeminal neuralgia)	14 (15) 9–25	3 (6) 1–17	0.17^a^
Type of contact	Contact caused by one or more arteries	38 (40) 30–51	28 (56) 44–74	0.10
	Contact caused by one or more veins	24 (24) 17–36	7 (14) 6–27	0.17
	Mixed contact (artery and vein)	19 (20) 12–29	12 (24) 14–40	0.73

The protocol and evaluation of the MRI scans were identical for patients with unilateral and bilateral pain. Results of the evaluations of patients with bilateral pain were included in the analysis of MRI findings only if the degree and type of contact between the trigeminal nerve and adjacent vessel(s), were the same on both sides. *Four patients with bilateral pain were excluded due to differences between sides. Four patients did not have a MRI according to protocol. **Displacement and/or atrophy of the trigeminal nerve. ^a^Fisher’s exact test. CL = 95% confidence limits

Changes in the pharmacological treatment, i.e. changes in dosages or types of drugs were carried out in 96 (93%) of the enrolled patients (initial visit compared to two-year assessment). Seven (7%) patients did not take any medication neither at baseline *nor* the two-year assessment. They continued their follow-up at the DHC due to the treatment of other coexisting headaches, primarily migraine and tension-type headache. At the initial assessment significantly more patients, 97 patients (85%), were using TN drugs, compared to 74 patients (72%) who were using TN drugs at their two-year assessment, (*p* < 0.001). The most frequently used drugs at the initial visit were carbamazepine (29 patients (28%)) and gabapentin (29 patients (28%)) (Table 6). At the two-year assessment, oxcarbazepine was the most frequently used drug (30 patients (29%)).

**Table 6:** Drugs used for trigeminal neuralgia at initial and follow-up assessments

	Initial assessment (*n* = 103) *	Dose Range (initial)	3 months assessment (*n* = 60)	6 months assessment (*n* = 71)	12 months assessment (*n* = 60)	Two-year assessment (*n* = 103)	Dose range (two-year)	*P*-value^#^
	*N* (%) Cl	*mg*	*N* (%) Cl	*N* (%) Cl	*N* (%) Cl	*N* (%) Cl	*mg*	
Carbamazepine	29 (28) 19–37	100–1600	22 (36) 24–47	22 (31) 21–43	15 (25) 14–37	20 (19) 12–28	200–1400	0.13
Oxcarbazepine	20 (19) 12–28	300–2400	20 (33) 22–46	18 (25) 16–37	18 (30) 19–43	30 (29) 21–39	300–2700	0.076
Gabapentin	29 (28) 20–37	600–4500	14 (23) 12–33%	17 (24) 14–36	12 (20) 10–32	24 (23) 15–32	600–3600	0.33
Pregabalin	8 (8) 3–13	150–600	7 (12) 3–19	2 (3) 0–9	6 (10) 4–21%	7 (7) 3–14	150–600	1.00
Baclofen	4 (4) 0–10	15–70	–	–	–	1 (1) 0–5	40	N/A
Valproate	4 (4) 0–10	500–1200	1 (2) 0–5	1 (1) 0–4	1 (2) 0–5	–	–	N/A
Tricyclic antidepressants	3 (3) 0–8	100–200	1 (2) 0–5	2 (2) 0–6	2 (3) 1–9	4 (4) 0–10	40–100	1.00
Lamotrigine	3 (3) 0–8	250–800	4 (6) 3–13	4 (6) 0–14	3 (5) 0–14	7 (7) 2–14	125–700	0.22
Phenytoin	1 (1) 0–5	50	–	–	–	–	–	N/A
Morphine-like drugs	2 (2) 0–7	200	–	–	–	2 (2) 0–7	200	N/A
Paracetamol	4 (4) 0–10	1000–4000	1 (2) 0–5	–	1 (2) 0–5	3 (3) 0–8	1000–4000	1.00
NSAID	2 (2) 0–7	400–1200	2 (3) 1–9	–	1 (2) 0–5	1 (1) 0–5	Md	N/A
Verapamil	1 (1) 0–7	480	–	–	1 (2) 0–5	–	–	N/A
Eslicarbazepine	–	–	–	–	–	1 (1) 0–7	800	N/A
Levetiracetam	–	–	–	–	–	1 (1) 0–7	1500	N/A

^a^Fishers exact test. *Represents the drugs that patients reported at their initial visit at DHC, i.e. the drugs they were using at the time of referral. ^#^Mc Nemars test based on data from initial vs. two-year assessment. CL = 95% confidence limits. *N/A* = not applicable, *Md* = missing data

The median dose of carbamazepine at the first visit was 600 mg (range 100–1600 mg) and 800 mg (range 200–1400 mg) at the two-year assessment, (*p* = 0.12). The median dose of oxcarbazepine at baseline was 1200 mg (range 300–2400 mg) and 900 mg (range 300–2700 mg) at the two-year assessment (*p* = 0.18). The median dose of gabapentin at baseline was 1600 mg (range 600–4500 mg) and 2100 mg (range 600–3600 mg) at the two-year assessment (*p* = 0.59) (Table 6).

Combination therapy with two or more drugs was used in 22 (21%) patients at baseline. The most frequent combinations were carbamazepine and gabapentin (4 patients), or oxcarbazepine and gabapentin (4 patients). At the two-year assessment 25 patients (24%) used combination therapy. The most frequently used combinations were oxcarbazepine and gabapentin (7 patients) and carbamazepine and gabapentin (6 patients). There was a significant increase in the number of patients who were not taking any TN drugs at the two-year assessment (31 patients (29%)) compared with baseline (17 patients (17%)), (*p* < 0.038).

During the follow-up period, the type or number of drugs was changed in 62 (60%) patients of whom the treatment was discontinued in 14 patients (23%), drug treatment was initiated in four patients (6%), while the type of drug was changed in 22 patients (36%). Combination therapy was altered to monotherapy in 6 patients (10%), combination therapy was initiated in 14 patients (23%), and combination therapy with three or more drugs was reduced to two drugs in 2 patients (3%).

### Patient satisfaction

The patient survey was returned by 52 (50%) patients. Of these, 45 patients answered the questions concerning satisfaction with the treatment and level of information given at the DHC. Regarding satisfaction concerning the treatment; 9 (20%) patients were unsatisfied, 4 (9%) patients were neither satisfied nor unsatisfied, while 32 (71%) patients were satisfied. Concerning the level of information: 6 (13%) patients were unsatisfied, 13 (13%) patients were neither satisfied nor unsatisfied, while 33 (73%) patients were satisfied.

### Lost to follow-up

Thirty-three patients (24%) were lost to follow-up. This was due to; a) Satisfied with current treatment and did not want further follow-up, *n* = 9, b) Did not have endpoint NRS recordings *n* = 6, c) Not satisfied with the treatment and did not want further follow-up, *n* = 4, d) Suffered from other diseases not related to TN (cancer, pregnant, pulmonary, urinary), n = 4, e) Dead not related to TN, *n* = 4, f) No out-patient visits, for unknown reasons, *n* = 2, g) Only interested in surgical treatment and discontinued follow up at DHC, *n* = 2, h) Moved to another country, *n* = 1, i) No further treatment options according to treating physician at 1st out-patient visit, *n* = 1.

### Non-pharmacological treatment

A standardised collection of data concerning the use of non-medical treatment have not been conducted in this study. However, we estimate that 10% of the patients have used the non-pharmacological treatment offers (physiotherapists or phycologists) and estimate that over 90% of the patients have talked to the specially trained nurses. Future studies are to be conducted.

## Discussion

This real-life prospective study demonstrates that TN patients enrolled in a structured multidisciplinary medical treatment program improve over two years with half of the patients having more than 50% reduction in the overall burden of pain. Findings are in line with a previous prospective study with a shorter follow-up (3–9.5 months), where 68% of the patients reported a 50% reduction of pain [7]. The reasons for this favourable prognosis are probably several; individualisation of drug treatment, optimisation of dose, education and support of the patients and appropriate referral of medically intractable patients to surgery. Natural history of the disease or regression towards the mean could play a role.

### Finding the right drug and dose for the individual patient

Our results could indicate that a structured program directed by specialists is effective. It is likely that individualised drug treatment and proper dose adjustments play a role. Almost all the enrolled patients underwent individual adjustment and/or change of drug during the time of follow-up. In the group of patients who were in current drug treatment, the dosage of medication was stable as well as there were no significant changes in the use of specific types of drugs. An increased number of patients were without drug treatment as well as there was an increment in the number of patients with *very good* or *good effect* of the drug treatment, during the follow-up.

### Non-pharmacological education and support

Although drug treatment is a cornerstone in TN management, patient-centered care including non-pharmacological therapy can improve treatment [24]. Furthermore, specialised pain centres offering multidisciplinary evaluation and care have shown to be highly efficacious and cost-effective [25]. We assume that the offered additional non-pharmacological support from specially trained nurses in-between follow-up visits, and support from psychologists, physiotherapists has had an influence on the effectiveness of the treatment. This has to be confirmed in future studies.

Our results show a more favourable prognosis of TN, than the prognosis of e.g. painful diabetic neuropathy. A recent study of treatment of painful diabetic neuropathy reported that only 30% of the patients, treated at a tertiary centre, reported a good outcome, defined as a 30% reduction of pain and 1-point reduction on the Pain Interference Scale [26]. Whether the differences in prognosis can be explained by the different etiologies, different study designs or the different pharmacological and non-pharmacological management has to be studied in future studies.

### Association between clinical characteristics and outcome

In a recent study, we found that the male sex and a neurovascular contact with morphological changes of the trigeminal nerve are strongly associated with a good outcome of microvascular decompression [10]. Based on these findings, we hypothesised that, in general, men have a monofactorial etiology (neurovascular contact) and women have a multifactorial etiology of TN. Interestingly, none of the clinical characteristics (apart from a long duration and co-existing chronic pain) was associated with a good outcome in the current study. These findings could reflect that the pathophysiology of all primary TN patients is the same (disregarding the etiology); hyperexcitable axons in the trigeminal nerve. Furthermore, the effects of medical treatment are also unrelated to etiology of the disease.

Our results do show that patients with coexisting chronic pain had poorer odds of a good outcome than patients without coexisting chronic pain. This is in line with previous studies demonstrating that complex patients with multiple pain conditions are more difficult to treat [27]. Another finding was the association between long disease duration and good outcome. Findings that contradicts what has previously been proposed; that TN becomes refractory to medical treatment, over time [2, 3]. However, since this was not the primary aim of the study, these results should be confirmed in other studies before final conclusions on these findings can be made.

### Natural history of the disease

Our results show that TN does not generally seem to progress. In our cohort, which might be expected to be the most difficult to treat, only a small subset of patients (apart from those referred to surgery) reported worsening of pain.

The current findings contradict the proposed natural history theory of TN as a progressive disorder that, over time will result in increased frequency and intensity of pain concomitant persistent pain and sensory disturbances [28, 29]. The results of Rasmussen and colleagues [6] also contradicts this theory of progressiveness as concomitant persistent pain did not seem to be a consequence of long-lasting paroxysmal pain; findings that we later on confirmed [30]. A recent clinical retrospective study also questions the theory, as only a minority of TN patients had worsening of pain over time, and only few patients became refractory to their drug treatment [4].

Our results could reflect that the natural history of TN is rather that of a non-progressive or slowly wearing off nature, or the results could reflect a regression towards the mean. To be able to make any final conclusions on the natural history of the disease, RCTs and even longer-term studies are needed. However, whatever the explanation for the current results, the findings do indicate that TN patients improve over a two-year period given that they are managed in a multidisciplinary setting directed by specialists, i.e. physicians, nurses, psychologists and physiotherapists.

### Relevant referral to neurosurgery

Our results are representative only for those TN patients who were medically managed and not referred for surgery. Fifty of those patients who were initially enrolled in the management program were referred to Department of Neurosurgery due to unsatisfactory management of their TN pain. We recently reported a favourable outcome for the selected group of TN patients who underwent a microvascular decompression at the Department of Neurosurgery [10]. The proportion of patients referred to neurosurgery was considered in line with what was recently reported by Zakrzewska et al., describing the utilization patterns of 3685 newly diagnosed TN patients [31].

Current TN guidelines recommend that if carbamazepine or oxcarbazepine treatment fails, patients are considered medically refractory and neurosurgical treatment is the next step [11]. The results of our study underline that optimisation of treatment should be done before patients are considered medically refractory. On the other hand, referral to neurosurgery should probably be done as soon as patients are considered medical refractory considering the severity of TN pain. The current study does not answer the important question regarding the optimal time for referral to surgery.

The patients with a poor outcome of the medical management in our cohort were all offered neurosurgical treatment, but many opted out of surgery. We hypothesise that it is either due to a relatively low overall burden of pain or fear of surgery, that holds the patients back for surgery. In Denmark healthcare is free why financial problems is not a barrier to surgery.

### Methodological considerations

The prospective design with standardised and systematic assessments of the patients and the set up as a real-life study in a clinical setting are the major strengths of this study. Real world data is difficult to acquire but does offer a more realistic view of the situation in the clinic due to fewer exclusion criteria. Although treatment practice was in line with international guidelines [11], choice of medical therapy and when to refer to neurosurgery invariably depends on the local tradition and patient preferences which probably varies according to culture. The project was embedded in a busy clinical setting and schemes to fill out were additional work for the consulting physicians. This might be the cause of some of the missing data.

The burden of overall pain was restricted to evaluate the patients’ evaluation of the average pain frequency and intensity, as opposed to a broader definition used by Tölle et al. [13]. It represents a limitation of the study that the BNI is a composite scale, why the interpretations of the results cannot be taken as a result of the individual units it is composed of (evaluation of pain and medical treatment).

This study is hypothesis-generating regarding the explanation of the favourable prognosis of TN. To be able to explain the causes of favourable prognosis, case-control studies or randomised controlled trials are required. Although randomising TN patients into subgroups where some patients are not given any preventative drugs or the neurosurgical procedure is postponed, would raise major ethical concerns. Future studies using a similar methodology from other specialist centres, as well as studies of even longer follow-up, are warranted to produce more real-life data and shed light on prognosis, treatment and the natural history of TN.

## Conclusion

This large prospective real-life study demonstrates that patients enrolled in a structured multidisciplinary TN management program at a specialist centre improve considerably over time. Thus, TN does not seem to be a disease with invariable progression in contrary to what has previously been assumed. The reasons for improvement could be several; optimisation of drug treatment, continuous advice and education and support by the multidisciplinary team, referral of the medically intractable patients for surgery or the natural history of the disease. The favourable prognosis provides hope and optimism for patients and care providers and suggests that specialist management of TN is highly rewarding.

## Clinical implications

• In TN patients who are managed in a multidisciplinary centre, the level of pain decreases over time and medication dosages remain stable

• Prognosis in medically treated TN patients is not dependent on concomitant persistent pain, neuroimaging findings or sex

• Findings indicate that TN is not an invariably progressive disease and provides optimism and hope to clinicians and patients that medical treatment of TN by experts is highly rewarding

## Additional files

Additional file 1: Supplementary material S1. Trigeminal neuralgia semi-structured interview. (DOCX 35 kb)

Additional file 2: Supplementary material S2 - Trigeminal neuralgia patient survey. (DOCX 27 kb)

Additional file 3: Supplementary material S3. Association between changes in the overall burden of pain and clinical characteristics. (DOCX 31 kb)

## Abbreviations

BNI: Barrow Neurological Institute pain scale
DHC: Danish Headache Center
ICHD: International Classification of Headache Disorders
NRS: Numeric Rating Scale
TN: trigeminal neuralgia
