# Supplementary material for: Favourable prognosis of trigeminal neuralgia when enrolled in a multidisciplinary management program - a two-year prospective real-life study
Source: J Headache Pain. 2019 Mar 4;20(1):23. doi: 10.1186/s10194-019-0973-4 (PMC6734423; doi:10.1186/s10194-019-0973-4)
Supplement: Supplementary file 1 — Supplementary material S1. Trigeminal neuralgia semi-structured interview. (DOCX 35 kb) [file 10194_2019_973_MOESM1_ESM.docx]

Supplementary material S1.

Trigeminal neuralgia semi-structured interview

Doctor (name):……… Date:…….…….

**1 Debut and course of TN:**

**1.1 Started with (type of pain):**

Stabbing pain: 1 Boring pain: 2

Debut, year: ……. Debut, year: ……..

**1.2 Year of diagnosis:** .................

**1.3.1 Trauma up to 6 months prior to debut?**

Yes 1 No 2

If yes, describe……………………………………………….,

**1.3.2 Has relevant imaging of face, jaw and teeth excluded other reasons for facial pain?**

Yes 1 No 2

**1.4 Has the pain changed from stabbing pain**

**to constant/boring pain:** Yes 1 No 2

Year :………….

**1.5 Does the patient experience any boring facial pain, after the debut of the stabbing pain (coexisting):**

Yes 1 No 2

What year :…………….

**1.6 Episodes of pain at night or during sleep?**

Yes 1 No 2

**1.7 Pain-free periods (+no medication needed):**

Yes 1 No 2 How long does a pain-free period last?

Months  1 Years  2

How many pain-free periods in total?..........................

**1.8 The global burden of pain over the last month - evaluated by the patient (frequency and intensity (NRS from 0- 10)):**

0 1 2 3 4 5 6 7 8 9 10

No burden extreme burden

**2.1 Stabbing pain (localization):**

Right Yes 1 No 2

Left 1 2

1. branch Yes 1 No 2

2. branch 1 2

3. branch 1 2

1.+2. branch 1 2

2.+3. branch 1 2

1.+2. +3. branch 1 2

Not anatomical distributed, 1 2

**2.1.2 On a scale from 0-10 where 0 is no pain and 10 is worst possible pain, how intense is the pain:** ...................

**2.2.1 Triggers of stabbing pain:** Yes No

Talking 1 2

Chewing 1 2

Brushing teeth 1 2

Cold wind 1 2

Touch 1 2

Spontaneous 1 2

Eating 1 2

Bright light 1 2

Brushing hair 1 2

Other 1 2

Describe: ………………………………………

*-----------PATIENTLABEL----------*

Name and date of birth

**2.2.2** **Trigger zones:**

Yes No

Right 1 2

Left 1 2

Yes No

1. branch 1 2

2. branch 1 2

3. branch 1 2

1.+2. branch 1 2

2.+3. branch 1 2

1.+2.+3. branch 1 2

**2.3.1 How long does one paroxysm of pain last:**

< 10 sec 1 10 sek- 2 mins 2

>2 mins 3

**2.3.2 Does the patients experience series of stabbing pain paroxysms:**

Yes 1 No 2

**2.3.3 How long does a series of paroxysms last:**

< 2 mins 1 2-60 mins 2 > 60 mins 3

**2.3.4 Number of daily attacks of stabbing pain (average over the last month):**

0 attack 0 6-10 attacks  4

1 attack 1 11-50 attacks  5

2 attacks 2 > 50 attacks  6

3-5 attacks  3

**3.1 Constant/Boring pain (localization):**

Right Yes 1 No 2

Left 1 2

1. branch Yes 1 No 2

2. branch 1 2

3. branch 1 2

1.+2. branch 1 2

2.+3. branch 1 2

1.+2. +3. branch 1 2

Not anatomical distributed, 1 2

**3.1.2 On a scale from 0-10 where 0 is no pain and 10 is worst possible pain, how intense is the pain:**………….

**3.2 Boring pain is worsened by:** Yes No

Talking 1 2

Chewing 1 2

Brushing teeth 1 2

Cold wind 1 2

Touch 1 2

Spontaneous 1 2

Eating 1 2

Bright light 1 2

Brushing hair 1 2

Other 1 2

Describe: ………………………………………

**3.3.1 Is the boring pain sporadic or constant:**

Sporadic 1 Constant 2

**3.3.2 If the pain is sporadic; indicate how often the pain is present during the wake hours of the day:** a. More than 50% Yes 1 No 2

b. Less than 50% 1 2

How many days per month (on average):……………

**4. Autonomic symptoms:** Yes No

Noticeable at every pain attack: 1 2 Tearing/conjunctival injection: 1 2

Nose running/clogged up: 1 2

Increased sweating: 1 2

Miosis or ptosis: 1 2

**5. Treatment**

**5.2 Previous prophylactic treatment:**

Efficacy Side effects

Yes No Yes No

a. Carbamazepine 1 2 1 2

b. Oxcarbazepine 1 2 1 2

c. Gabapentin 1 2 1 2

d. Other……………………… 1 2 1 2

e. Other ……………………… 1 2 1 2

**5.3** **Efficacy of current prophylactic drug treatment (relevant for patients in prophylactic treatment):**

I Very good efficacy: *no pain*

II Good effect: *Occasional pain that does not, or only occasionally, reduce my quality of life*

III Limited effect: *Daily pain with moderate reduction of my quality of life*

IV Insufficient effect: *Daily episodes with severe pain which significantly reduce my quality of life*

**5.4** **Side-effects from current prophylactic drug treatment:**

***0****: No side effects;* ***1****: Mild (does not affect daily work/ activity);* ***2****: moderate (Effects but does not obstruct daily work/activity);* ***3****: severer (Obstructs daily work/activity)*

Side effects: Daily dosis:

a. Carbamazepine **0** **1** **2** **3** ………………

b. Oxcarbazepine **0** **1** **2** **3** ……………….

c. Gabapentin **0** **1** **2** **3** ……………….

d. Other ………… **0** **1** **2** **3** ……………….

**5.5 Patient satisfaction with current treatment and situation:**

Very unsatisfied-Unsatisfied-Satisfied-Very satisfied

Pain 1 2 3 4 5 6 7

Side-effects 1 2 3 4 5 6 7

Level of info. 1 2 3 4 5 6 7

###### 6. Priveous treatment for facial pain: Yes No

###### a. Root canal treatment 1 2

b. Dental extraction 1 2

c. Antibiotics for sinusitis 1 2

d. Sinus-surgery 1 2

e. Puncture of sinuses 1 2

f. Chiropractor 1 2

d. Migraine treatment 1 2

###### e. Cluster headache treatment 1 2

###### f. Cranio-sacral therapy 1 2

g. Glycerol nerve block 1 2

h. Microvascular decompression 1 2

i. Rhizotomy 1 2

###### j. Balloon compression 1 2

###### k. Other 1 2

###### Describe:………………………………

**7. Comorbidity**  Yes No

a. Concussion 1 2

b. Skull fracture 1 2

c. Encephalitis 1 2

d. Meningitis 1 2

e. Brain tumour 1 2

f. Epilepsy 1 2

g. Ischemic infarction 1 2

h. Brainstem infarction 1 2

i. Brain haemorrhage 1 2

j. TCI 1 2

k. Hypertension 1 2

l. Other cardiovascular diseases 1 2

m. Anxiety 1 2

n. Depression 1 2

o. Facial trauma/fracture 1 2

p. Multiple Sclerosis 1 2

q. Sinusitis 1 2

r. Sinus-tumour 1 2

s. Rhino-pharynx tumour 1 2

t. Other chronic pain diseases

which lasted more than 3 months 1 2

u. Whiplash 1 2

**8. Other headache:** Yes No

a. Cluster headache 1 2

b. Migraine 1 2

c. Tension type headache 1 2

d. Other headache (ICHD):……………………….

**10. Negative effects on physical health/decrease of QOL:**

Yes No Not relevant

Work 1 2 3

Education 1 2 3

Leisure time 1 2 3

**11. The influence on psychiatric health:**

Have you experienced (over the last month): Yes No

- feeling “down”, hopeless, depressed? 1 2

- decreased interest in doing things you

normally like? 1 2

###### 13. Diagnosis (ICHD-III beta) Yes No

a.(13.1.1.1) Trigeminal neuralgia,

only paroxysmal 1 2

b. (13.1.1.2) Trigeminal neuralgia

with concomitant persistent pain 1 2

c. (13.1.2.4) Painful trigeminal

neuropathy due to MS plaque at the root of

the trigeminal nerve 1 2

d. (13.1.2.5) Painful trigeminal

neuropathy due to space-occupying lesion 1 2

e. (13.1.2.3) Painful posttraumatic

trigeminal neuropathy 1 2

f. (13.11) Persistent idiopathic

facial pain 1 2

g. (13.12.1) Central neuropathic pain

due to MS 1 2

h. Possible trigeminal neuralgia 1 2

i. Other diagnosis:…………………….
