# Supplementary material for: Favourable prognosis of trigeminal neuralgia when enrolled in a multidisciplinary management program - a two-year prospective real-life study
Source: J Headache Pain. 2019 Mar 4;20(1):23. doi: 10.1186/s10194-019-0973-4 (PMC6734423; doi:10.1186/s10194-019-0973-4)
Supplement: Supplementary file 2 — Supplementary material S2 - Trigeminal neuralgia patient survey. (DOCX 27 kb) [file 10194_2019_973_MOESM2_ESM.docx]

Supplementary material S2 - Trigeminal neuralgia patient survey

| **Name:__________________________________________________________________**  **Date of birth:_______________________-____________Current date:____/____ - ________** |
| --- |
| 1. **Global burden of pain (including the frequency and intensity)** 2. Indicate how high the total burden of your facial pain (including frequency and intensity) has been over the last month (encircle your answer)?   0 1 2 3 4 5 6 7 8 9 10  No burden extreme burden   1. Over the last two years, have you had any pain-free periods (no pain and no drugs where needed to treat your trigeminal neuralgia?   Yes No (please proceed to question nr 2)   1. If yes, how long did the pain-free period last (average if more than one)?   Months Years   1. How many pain-free periods have you had over the last two years?   1 4  2 5  3 More than 5 |
| 1. **Stabbing pain - as you experience it right now (average over the last week)** 2. How painful have your trigeminal neuralgia pain been on average over the last week. (Only describe the stabbing/jabbing pain) (encircle your answer)?     0 1 2 3 4 5 6 7 8 9 10  No pain the worst ever   1. How many pain-attacks have you had, on average, per day?   0 attacks 1 attacks 2 attacks 3-5 attacks  6-10 attacks 10-50 attacks More than 50 attacks |
| 1. **Boring/ dull pain - as you experience it right now (average over the last week)**   Some trigeminal neuralgia patients do not only experience short-lasting stabbing pain, but may also experience a boring, dull, more persistent pain in the same area as the stabbing pain occurs.   1. Do you experience boring/dull facial pain? Yes No (please proceed to question nr.4) 2. How painful have your boring/dull pain been over the last week (on average)? (Only describe the boring/dull pain) (encircle your answer).     0 1 2 3 4 5 6 7 8 9 10  No pain the worst ever |
| 1. **Current drug treatment for trigeminal neuralgia** 2. Do you currently take any drugs for your trigeminal neuralgia?   Yes No (please proceed to question nr 5)   1. Please list the drugs that you currently use to treat your trigeminal neuralgia:  \| Name of the drug \| Daily dose in milligram (i.e. if you are prescribed 400 mg two times per day, then the daily dose = 800 mg) \| \| --- \| --- \| \|  \|  \| \|  \|  \| \|  \|  \|  1. How well do you think the drug works?   Very good effect: *No pain*  Good effect: *Occasional pain that does not, or only occasionally, reduce my quality of life.*  Limited effect: *Daily pain with a moderate reduction my quality of life*  Insufficient effect: *Daily episodes with severe pain which significantly reduce my quality of life*   1. How many side-effects do you experience from current treatment?   No side-effects  Only few side-effects that does not reduce my ability to work or do any normal daily activities  Moderate side-effects, that decreases my quality of life a little but does not hinder me from  working or other daily activities.  Many side-effects that hinder me from working and/or doing other daily activities |
| 1. **The disease’ effect on daily living and activities** 2. Do you experience that the pain hinders you from doing daily activities?   Regarding work? yes No Not relevant  Regarding education? yes No Not relevant  Regarding hobbies and spare time activities? yes No   1. Over the last month, have you felt depressed, ”down” or hopeless? yes No 2. Over the last month, have you been lacking interest of things you usually liked and wanted to do? yes No |
| 1. **Level of satisfaction with treatment and information at the Danish Headache Center**   How satisfied are you with the current treatment compared to the treatment that you were offered before you were enrolled in the treatment regime at the Danish Headache Center?   1. Regarding your trigeminal neuralgia pain (encircle your answer)?   Very unsatisfied - Unsatisfied - Satisfied - Very satisfied  1 2 3 4 5 6 7   1. Regarding the side-effects of your trigeminal neuralgia related drug treatment (encircle your answer)?   Very unsatisfied - Unsatisfied - Satisfied - Very satisfied  1 2 3 4 5 6 7   1. Regarding the level of information given (encircle your answer)?   Very unsatisfied - Unsatisfied - Satisfied - Very satisfied  1 2 3 4 5 6 7 |

THANK YOU - because you completed the patient survey. If you have any comments or suggestions on how the Danish Headache Center can improve the treatment of trigeminus neuralgia, please write them below.

|  |  |  |  |
| --- | --- | --- | --- |
|  |  |  |  |
|  |  |  |  |
|  |  |  |  |
|  |  |  |  |
|  |  |  |  |
|  |  |  |  |
|  |  |  |  |
