# Supplementary material for: Favourable prognosis of trigeminal neuralgia when enrolled in a multidisciplinary management program - a two-year prospective real-life study
Source: J Headache Pain. 2019 Mar 4;20(1):23. doi: 10.1186/s10194-019-0973-4 (PMC6734423; doi:10.1186/s10194-019-0973-4)
Supplement: Supplementary file 3 — Supplementary material S3. Association between changes in the overall burden of pain and clinical characteristics. (DOCX 31 kb) [file 10194_2019_973_MOESM3_ESM.docx]

**Supplementary material S3.**

**Association between changes in the overall burden of pain and clinical characteristics**

N = 103. Distribution of outcome in subgroups with specific clinical characteristics. Number presented in ( ) represent numbers of patients with the specific characteristic. * = Chi^2^ test, p < 0.05.
